# Gut Microbial Metabolite Short-Chain Fatt Acids Partially Reverse Surgery and Anesthesia-Induced Behavior Deficits in C57BL/6J Mice

**DOI:** 10.3389/fnins.2021.664641

**Published:** 2021-06-08

**Authors:** Xiaohan Xu, Kexin Wang, Xuezhao Cao, Zhe Li, Yongjian Zhou, Jiancong Ren, Fang Liu

**Affiliations:** ^1^Department of Anesthesiology, The First Affiliated Hospital of China Medical University, Shenyang, China; ^2^Department of Thyroid Surgery, The First Affiliated Hospital of Zhengzhou University, Zhengzhou, China; ^3^Department of Neurology, The First Affiliated Hospital of China Medical University, Shenyang, China

**Keywords:** gut microbiota, neuroinflammation, surgical trauma and anesthesia, perioperative neurocognitive disorders, short-chain fatty acids

## Abstract

Accumulating evidence has demonstrated that damages of gut microbiota are strongly associated with central nervous system (CNS) diseases, such as perioperative neurocognitive disorders (PND). The present study investigated the role of gut microbial metabolite short-chain fatty acids (SCFAs) in surgery-induced cognitive deficits and neuroinflammation in the hippocampus. Adult male C57BL/6J mice received either SCFA mixture or saline orally for 4 weeks, and then partial hepatectomy was performed. The fecal supernatant of surgical mice was transplanted to normal mice for 3 weeks. The Morris water maze (MWM) and open-field tests were used to evaluate behavioral performance on postoperative or post-transplantation days 3 and 7. In the MWM test, pretreatment with exogenous SCFAs partially reversed surgery-induced impairments in crossing times and the time spent in the target quadrant on postoperative day 3 (*p* < 0.05, *p* < 0.05, respectively). In the open-field test, compared with the surgical mice, exogenous SCFA administration prior to surgery partially improved the locomotor activity (*p* < 0.05) and anxiety-like behavior (*p* < 0.05) on postoperative day 3. Surgical trauma and anesthesia enhanced ionized calcium-binding adapter molecule 1 (Iba-1) expression (*p* < 0.001), increased the levels of interleukin (IL)-1β (*p* < 0.001) and IL-6 (*p* < 0.001), and inhibited SCFA production (*p* < 0.001) on postoperative day 3. The expression of the brain-derived neurotrophic factor (BDNF) was also decreased (*p* < 0.001). Overall, surgical trauma and anesthesia exacerbated cognitive impairment, enhanced neuroinflammatory responses, and inhibited SCFA production. Pretreatment with SCFAs attenuated these effects partially by reversing microglial overactivation, inhibiting neuroinflammatory responses, and enhancing BDNF expression.

## Introduction

Perioperative neurocognitive disorders (PND), characterized by reversible impairment in memory, mental concentration, is one of the most common postoperative complications in elderly patients. PND may result in delayed recovery, prolonged hospitalization, and an increased risk of disability and mortality (Cao et al., 2010). However, the neurobiological basis of PND remains unknown. Advanced age, multiple surgeries, and duration of anesthesia have been implicated as the risk factors for PND. Accumulating evidence has suggested that neuroinflammation plays a crucial role in the pathogenesis of PND (Steinmetz et al., 2009; Li Z. et al., 2018). Our previous studies indicated that surgical trauma and anesthesia induced microglial activation, leading to cytokine cascade activation and inflammatory mediator release in the hippocampus (Wang et al., 2017; Jiang et al., 2018; Zhou et al., 2019).

A growing body of evidence manifests that gut microbiota can remotely regulate brain function and behavior by neural, immune, and endocrine pathways of the brain–gut–microbiota axis (Kelly et al., 2015). Yu et al. (2019) observed that the gut microbial composition of elderly mice is very different after surgery and anesthesia. The potential contribution of bidirectional communication between the gut and brain to human health has been revealed by the association between gastrointestinal and mood disorders. Emerging evidence indicates that the microbiota–gut–brain axis plays an important role in cognitive function. The impaired composition of gut microbiota may be a contributor to the exaggerated microglial activation, neuroinflammatory responses, and behavioral deficits in response to surgery challenges.

Gut microbial metabolism is known to produce catecholamines, histamine, and/or other neuroactive mediators that can directly stimulate the local enteric nervous system and/or primary afferent fibers of vagal or dorsal root origin. In addition to direct interactions with neural processes, immune activation and inflammation participate in nearly all neurologic/psychiatric disorders (Duca et al., 2014; Lyman et al., 2014), and gut dysbiosis might alter brain function via this pathway (Bruce-Keller et al., 2015). An abnormal composition of gut microbiota has been observed to be greatly associated with the onset of Alzheimer’s disease (AD), autism, schizophrenia, and depression (Lv et al., 2017; Yang et al., 2017a, b; Zhan et al., 2018). Consequently, these results suggest that gut microbiota, at least partially, is associated with the pathogenesis of brain disorders (Kelly et al., 2016; Rezaei Asl et al., 2019).

Neuroinflammatory response triggered by surgery and anesthesia is a major contributor to the development of PND. Therefore, modulation of cytokine-mediated cytotoxicity is expected to be a new therapeutic strategy for PND. Recent evidence suggests that products of normal gut microbiota, such as SCFAs, might influence immune responses positively and prevent the occurrence and development of inflammatory diseases (Mazmanian et al., 2008; Wen et al., 2008; Smith et al., 2013). The physiological effects of SCFAs have been well-documented, which reduce the production of proinflammatory cytokines (Ni et al., 2010) and have a well-characterized anti-inflammatory effect (Cox et al., 2009; Vinolo et al., 2011). Three main types of SCFAs (acetate, propionate, and butyrate) decreased the levels of proinflammatory cytokines (e.g., IL-1β, IL-6, tumor necrosis factor (TNF)-α, and nitric oxide (NO)), inhibiting the vitality of inducible-nitric oxide synthase (iNOS) as well as enhancing the production of anti-inflammatory cytokine IL-10 in lipopolysaccharide (LPS)-stimulated macrophage-like RAW264.7 cells. Cox et al. (2009) also confirmed that SCFAs inhibited LPS-induced production of interferon (IFN)-γ and TNF-α in human peripheral blood mononuclear cells. SCFAs have been used to treat inflammatory conditions in human and animal models, which may be involved in inflammatory cytokine-mediated PND.

Microglia, the resident immune cells in the brain, are essential for regulating neuroinflammation, influencing synaptic remodeling, and modulating neurogenesis. Microglial dysfunction has been implicated in the onset and progression of several neurodegenerative diseases and psychiatric disorders (Abdel-Haq et al., 2019). Mounting evidence indicates that microglial overactivation contributes to neuronal damage in neurodegenerative diseases (Block et al., 2007; Wang et al., 2016). The gut microbiota has been indicated to significantly influence microglial activation and gut-derived microbial metabolites (e.g., SCFAs) regulate neuroinflammatory response mediated by microglia (Cox et al., 2009), which inspired our hypothesis that microglia may be a critical mediator between the gut microbiome and PND.

The role of gut microbiota in PND is largely unknown. SCFAs, the metabolites generated by gut microbiota, have been implicated in gastrointestinal function (neuro)immune regulation, and host metabolism (Maslowski et al., 2009; van de Wouw et al., 2018), but their role in stress-induced behavioral and physiological alterations is poorly understood. It is known that surgical trauma and anesthesia altered the composition of gut microbiota (Zheng et al., 2016). The main objective of this study was to investigate whether gut microbiota plays a critical role in behavioral deficits and neuroinflammatory responses following a major surgical intervention. Furthermore, the study aimed to investigate whether SCFA supplementation could ameliorate surgical trauma and anesthesia-induced behavioral deficits by inhibiting proinflammatory cytokine expression, modulating microglial activation, and enhancing BDNF expression in the brains of adult mice. Thus, the manipulation of gut microbiota may be a useful tool to decode the role of SCFAs in prevention and treatment of PND.

## Materials and Methods

### Animals

Adult (8–10 weeks old) male C57BL/6J mice were randomly divided into five groups: control group (*n* = 30), SCFA group (*n* = 30), surgery group (*n* = 30), SCFAs + surgery group (*n* = 30), and fecal microbiota transplantation (FMT) group (*n* = 30). All animals were group-housed in a temperature-controlled (23 ± 1°C) room on a 12-h light and dark cycle with *ad libitum* access to food and water.

### Experimental Procedure

**FIGURE 1:**
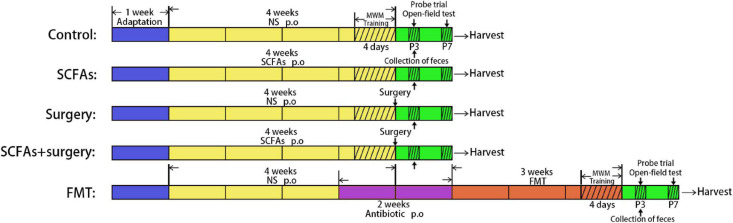
The schedule of the present study.

All the mice were allowed to acclimate for 1 week before the experiment. The animals in the SCFA group and SCFAs + surgery group were orally gavaged with SCFA mixture (the main components are sodium acetate (67.5 mM), sodium propionate (25 mM), and sodium butyrate (40 mM), dissolved into the drinking water (Yuanye Biotechnology Co. Ltd, Shanghai, China, PH = 7.6) for 4 weeks and refreshed three times per week. The mice in the surgery group and SCFAs + surgery group were subjected to partial hepatectomy on the last day. Partial hepatectomy was performed under general anesthesia (a gas mixture of 1.5–2.5% isoflurane (Baxter, United States) and oxygen at 2 L/min). The liver was exposed through a 1–2-cm midline abdominal incision. The left lateral lobes of the liver (approximately 30% of the organ) were excised. To limit variability, all surgeries were performed by the same person. Microbiota was freshly harvested from the cecum of surgical mice on postoperative day 3. The mice in the FMT group received broad-spectrum antibiotics (ampicillin 1 g/L, neomycin sulfate 1 g/L, and metronidazole 1 g/L (Sigma-Aldrich, Shanghai, China) dissolved in drinking water) orally once daily for 2 consecutive weeks prior to the transplantation. The drinking solution was renewed every 2 days. To recolonize the gut of fecal microbiota transplantation mice, the recipient mice were orally gavaged with 200 μL of cecal content isolated from surgical mice over the subsequent 3 weeks (3 days per week, twice a day). Fecal samples in all groups were harvested following behavioral tests on postoperative and post-transplantation day 3 and immediately stored at −80°C for further processing and gas chromatography–mass spectrometry (GC-MS) [Agilent 7890B/7000D (Agilent Technologies, Santa Clara, CA, United States)]. All mice were trained using the MWM test for four times per day for 4 consecutive days before the surgery procedure or fecal microbiota transplantation. Behavioral performances were evaluated using the MWM test and open-field test on postoperative or post-transplantation days 3 and 7. The mice were sacrificed after behavioral tests at each time point, and the brain was rapidly harvested for biochemical analyses (Figure 1). All procedures were conducted in accordance with the Declaration of the National Institutes of Health Guide for Care and Use of Laboratory Animals and approved by the China Medical University Animal Care and Use Committee (CMU2019261).

### Behavioral Tests

Spatial learning and memory were evaluated using an MWM test. Pool temperature was maintained at 25 ± 0.5°C. The mice were trained with the platform in a fixed location for four times per day for 4 consecutive days. On postoperative days 3 and 7, mice were subjected to a probe trial in which the platform was removed and the visual cues remained. A video camera with a computerized animal tracking system (Version 2.1 Beijing Sunny Instruments Co. Ltd, Mouse Track Viewer) was used to monitor swimming speed, distance, and latency to the platform. All testing was conducted at roughly the same time each day in order to minimize variability in performance due to time of day. The behavior performance was assessed by an investigator who was unaware of the intervention condition. To assess anxiety-like behavior and spontaneous locomotor ability, an open-field test was performed on postoperative or post-transplantation days 3 and 7. The open-field observation cage consists of a square wooden arena (40 × 40 × 40 cm) with its inside walls covered in black. The mice were placed in the center of the area and allowed to explore freely. All spontaneous activities were recorded for 5 min using a video tracking software (Smart, San Diego Instruments, San Diego, CA, United States). Total distance traveled and time spent in the central area were recorded. The test was conducted in a quiet room and indirect lighting in the morning (8:00–12:00 A.M.). The floor surfaces and walls of the apparatus were thoroughly cleaned between each test with 50% alcohol solution so that the animals were not affected by the odors of the previous urine and feces.

### Western Blot Analysis

Hippocampal tissue samples were homogenized in ice-cold strong Radio-Immunoprecipitation Assay (RIPA) lysis buffer (Beyotime, Shanghai, China) containing 1% Protease Inhibitors (Cell Signaling Technology, Danvers, MA). Then, the lysates were shaken in an ice bath for 30 min and cleared by centrifugation (12,000 rpm, 10 min, 4°C). The supernatant was collected. Protein concentrations were determined using the bicinchoninic acid (BCA) protein assay kit (Beyotime, Shanghai, China). The normalized protein samples were denatured by boiling for 5 min. Equal amounts of tissue samples were transferred to polyvinylidene fluoride (PVDF) microporous membranes, which were then blocked with 5% skim milk in Tris-buffered saline Tween20 (TBST) at room temperature for 2 h. Membranes were then incubated using the following antibodies: anti-IL-1β (1:2,000; Abcam, Cambridge, United Kingdom), anti-IL-6 (1:2,500; Abcam, Cambridge, United Kingdom), and anti-BDNF (1:2,000; Abcam, Cambridge, United Kingdom) overnight at 4°C. After washing with TBST, the membranes were then incubated in appropriate secondary antibodies (anti-mouse and anti-rabbit, 1:10,000) diluted in TBST for 2 h at room temperature. The protein bands were visualized by using an enhanced chemiluminescence detection kit (ECL Advance Kit; Bio-Rad) and Image Lab software (Image Lab). The relative expression levels were calculated as the intensity ratio of target protein to β-actin (ZSGB-BIO, Beijing, China) or GAPDH (Proteintech, Wuhan, China).

### Immunohistochemistry

The mice were sacrificed by cervical dislocation. The brain tissue of the mouse was harvested quickly, and the following process was performed on ice. The hippocampus tissue of one half of the brain was quickly dissected and stored at −80°C for the subsequent Western blotting experiment. The other hemispheres of all mice were fixed in paraformaldehyde and transferred to PBS buffer after 24 h for the immunohistochemistry experiment. Wax sections (5 μM) of the hippocampus were deparaffinized, rehydrated, and incubated with 3% H_2_O_2_ in methanol at room temperature for 10 min to block endogenous peroxidase. After washing three times with phosphate-buffered saline (PBS), the sections were then incubated in PBS overnight at 4°C with goat-anti ionized calcium-binding adapter molecule 1 (Iba-1; 1:200; Abcam, Cambridge, United Kingdom). Then, the sections were incubated with 1:500 rabbit-anti goat secondary antibody (Jackson ImmunoResearch, Wet Grove, PA) for 20 min. All sections were then incubated for 20 min with the avidin–biotin peroxidase complex (Vectastain ABC kit, Vector, Burlingame, CA) at room temperature. After being thoroughly washed, the sections were visualized using the DAB method. The reaction products were then counterstained with hematoxylin, dehydrated, mounted, and visualized using a compound light microscope. For quantification, the positive cells in the hippocampus were estimated by using the mean integrated optical density (IOD) and analyzed using image analysis software (Image Pro Plus 6.0).

### Gas Chromatography–Mass Spectrometry

GC-MS was used to evaluate the SCFA expression in gut microbial metabolites in surgical mice (Zhai et al., 2018). The samples (100 ± 1 mg) were extracted with 1 mL dH_2_O and vortex mixing for 10 s. Then, the samples were homogenized in a ball mill for 4 min at 45 Hz, ultrasound treated for 5 min (incubated in ice water), and then centrifuged at 5,000 rpm, 4°C, for 20 min. The supernatant (0.6 mL) was transferred into fresh 2-mL EP tubes, containing 0.1 mL 50% H_2_SO_4_ and 0.5 mL of 2-methylvaleric acid (50 μg/mL stock in methyl tert-butyl ether) as internal standard. Then, after oscillating extraction for 10 min, it was ultrasound treated for 10 min (incubated in ice water) and centrifuged for 10 min at 12,000 rpm, 4°C. Specific analysis conditions of GC-MS are as follows: GC-MS analysis was performed using an Agilent 7890 gas chromatograph system coupled with an Agilent 7000D mass spectrometer. The system utilized a HP-FFAP capillary column. A 1-μL aliquot of the analyte was injected in split mode (5:1). Helium was used as the carrier gas, the front inlet purge flow was 3 mL/min, and the rate of gas flow through the column was 1 mL/min. The initial temperature was kept at 100°C for 1 min, then raised to 145°C at a rate of 5°C/min and finally to 240°C at a rate of 20°C/min. The injection, transfer line, quad, and ion source temperatures were 250, 280, 150, and 230°C, respectively. The energy was -70 eV in electron impact mode. The mass spectrometry data were acquired in full-scan mode with the m/z range of 20–400 after a solvent delay immediately.

### Statistical Analysis

The Statistical Package for the Social Sciences (SPSS) 25.0 and GraphPad Software 8.0 statistical software (Prism, Inc., La Jolla, CA, United States) was used for statistical analyses. The data are presented as mean ± standard error of the mean (SEM). One-way analysis of variance (ANOVA) was utilized in comparisons more than two groups. The training behavioral parameters were analyzed by a repeated two-way ANOVA. A post-test was employed when ANOVA demonstrated a statistically significant difference. *p* < 0.05 was considered statistically significant.

## Results

### Pretreatment With SCFAs Ameliorated Surgical Trauma and Anesthesia- Induced Behavioral Deficits

**FIGURE 2:**
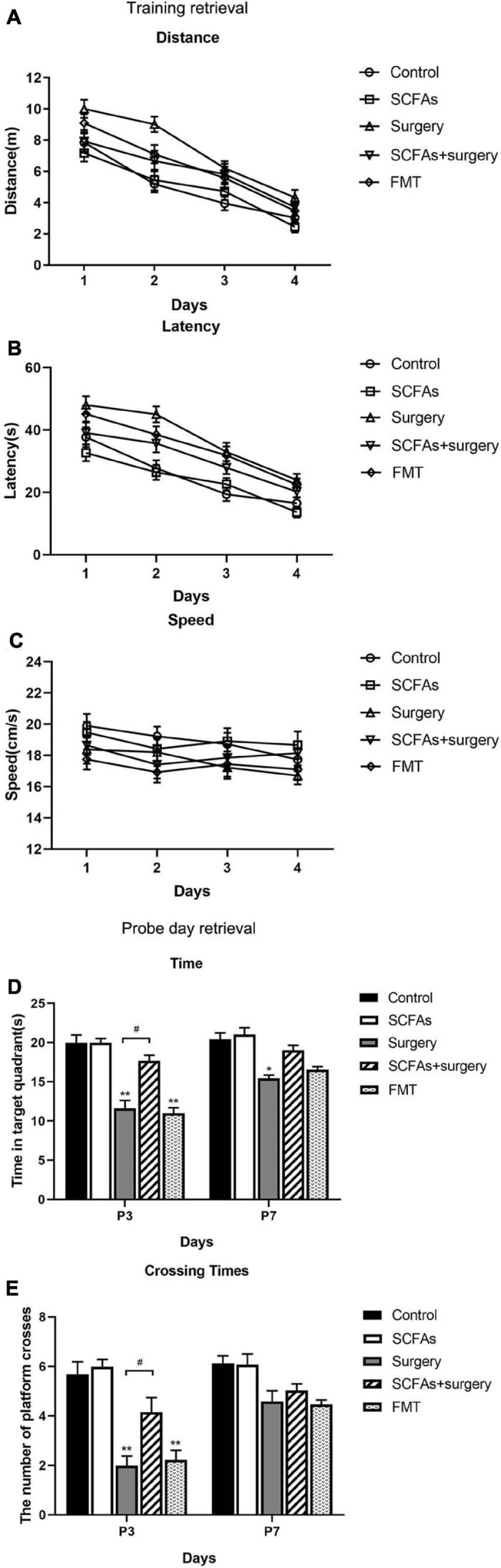
All mice showed improvements in swimming latency and distance over 4 consecutive training days in a Morris water maze (MWM). The mice in the SCFAs + surgery group performed better (swam less distance and less latency) than those in the surgery group on training days 2 and 3. Surgical trauma and anesthesia and fecal microbiota transplantation significantly exacerbated spatial learning and memory impairment in the probe trial test compared with the controls on postoperative or post-transplantation day 3. **(A)** Distance traveled to the platform. **(B)** Escape latency to the platform. **(C)** Swimming speed. **(D)** Time spent in target quadrant. **(E)** Cross platform times. Bars represent mean ± SEM. *p < 0.05 vs. the day-matched control group, **p < 0.001 vs. the day-matched control group, ^#^*p* < 0.05 vs. the day-matched surgery group. P3 and P7: postoperative or post-transplantation days 3 and 7, respectively.

**FIGURE 3:**
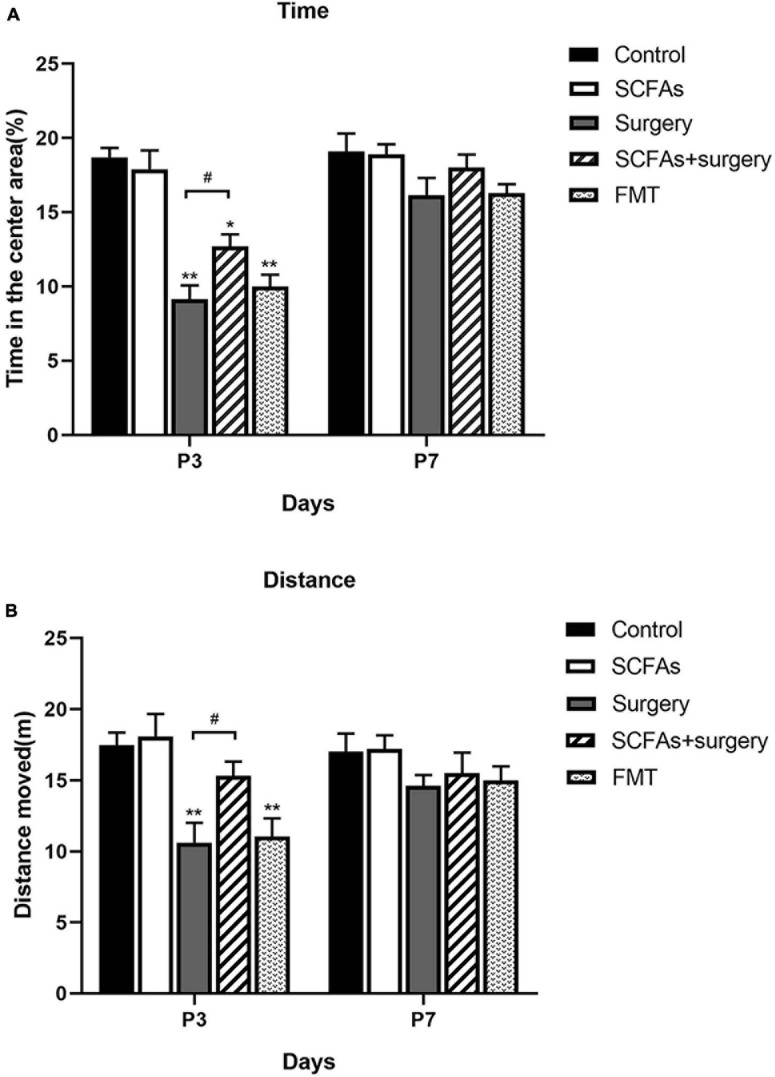
Surgical trauma and anesthesia and fecal microbiota transplantation exacerbated neurobehavioral impairment on postoperative or post-transplantation day 3 in the open-field test. Treatment with SCFAs significantly improved behavioral performance on postoperative day 3. **(A)** The time spent in the center area. **(B)** Total distance moved in the center. The results are represented as mean ± SEM. *p < 0.05 vs. the day-matched control group, **p < 0.001 vs. the day-matched control group, ^#^*p* < 0.05 vs. the day-matched surgery group. P3 and P7: postoperative or post-transplantation days 3 and 7, respectively.

The MWM test indicated that all animals showed improvements in spatial learning and memory over 4 consecutive training days. Repeated measures of ANOVA of swimming data revealed significant effects of surgery, exogenous SCFA administration, and day on both the distance and latency (Figures 2A,B) but not on the speed (Figure 2C). A post-test showed that surgical trauma, anesthesia, and fecal microbiota transplantation significantly decreased the time spent in target quadrant compared with the control group on postoperative or post-transplantation day 3 (*p* < 0.001, *p* < 0.001) and improved on day 7 (*p* > 0.05, *p* > 0.05) in the probe trial test (Figure 2D). Similarly, the times crossing the targeted zone were significantly reduced in surgical and FMT mice compared with the controls on postoperative or post-transplantation day 3 (*p* < 0.001, *p* < 0.001) and improved on day 7 (*p* > 0.05, *p* > 0.05) (Figure 2E). Furthermore, compared with surgical mice, pretreatment with exogenous SCFAs partially reversed the impairments in crossing times and the time spent in the target quadrant in the SCFAs + surgery group on postoperative day 3 (*p* < 0.05, *p* < 0.05). There was no significant difference in average swimming speed (*p* > 0.05) among groups, which suggests that the poorer performance of surgical mice did not result from a lack of motivation or reduced motor ability. Spontaneous locomotor activity and anxiety-like behavior were assessed in the open-field test. Two-way ANOVA of the total distance traveled and the time in the central area revealed significant effects of surgery and exogenous SCFA administration on postoperative day 3. A post-test showed that surgical trauma and anesthesia significantly reduced the time spent in the central area compared with the controls on postoperative day 3 (*p* < 0.001) and recovered on day 7 (*p* > 0.05) (Figure 3A). The total distance traveled of surgical mice was significantly decreased compared with the control group on postoperative day 3 (*p* < 0.001) and improved on day 7 (*p* > 0.05) (Figure 3B). Compared with the surgical mice, exogenous SCFA administration prior to surgery partially improved the locomotor activity (*p* < 0.05) and anxiety-like behavior (*p* < 0.05) on postoperative day 3.

### Surgical Trauma and Anesthesia Exacerbated Hippocampal Neuroinflammatory Responses and Pretreatment With SCFA-Downregulated IL-1β and IL-6 Expression

**FIGURE 4:**
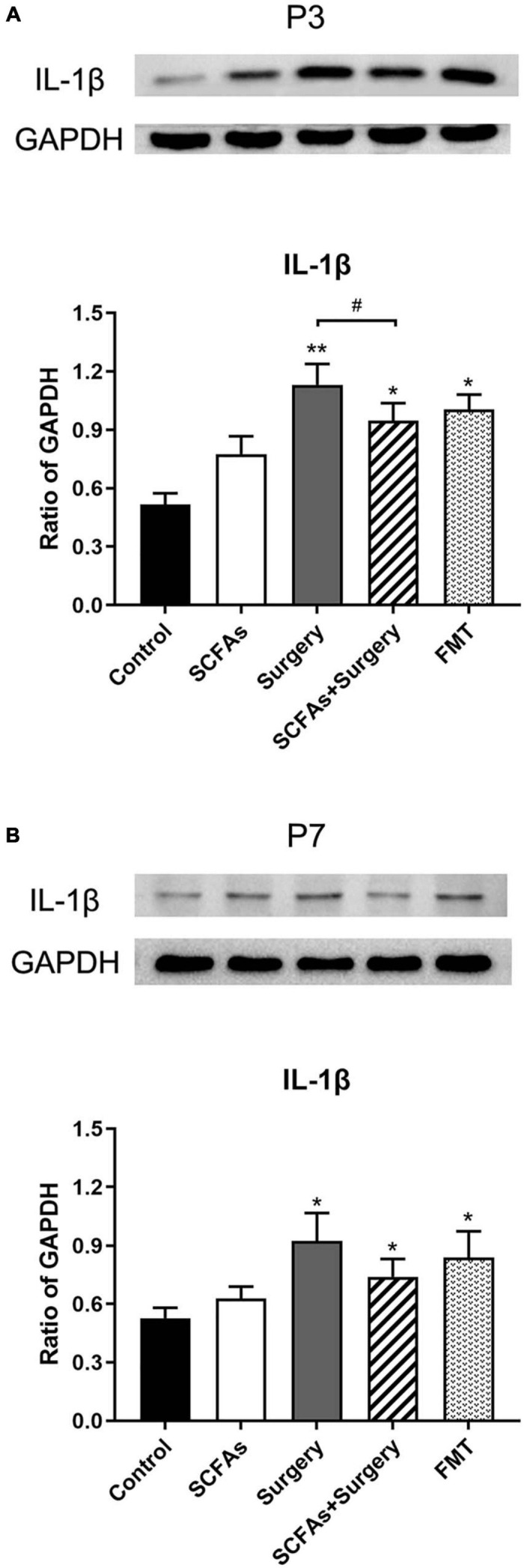
The level of hippocampal IL-1β was increased following the surgery procedure and fecal microbiota transplantation on postoperative or post-transplantation day 3 compared with the control group. SCFA pretreatment partially impeded the increased level of IL-1β compared with the surgery group on postoperative day 3. **(A)** The level of hippocampal IL-1β on postoperative or post-transplantation day 3. **(B)** The level of hippocampal IL-1β on postoperative or post-transplantation day 7. The results are represented as mean ± SEM. *p < 0.05 vs. the day-matched control group, **p < 0.001 vs. the day-matched control group, ^#^*p* < 0.05 vs. the day-matched surgery group. P3 and P7: postoperative or post-transplantation days 3 and 7, respectively.

**FIGURE 5:**
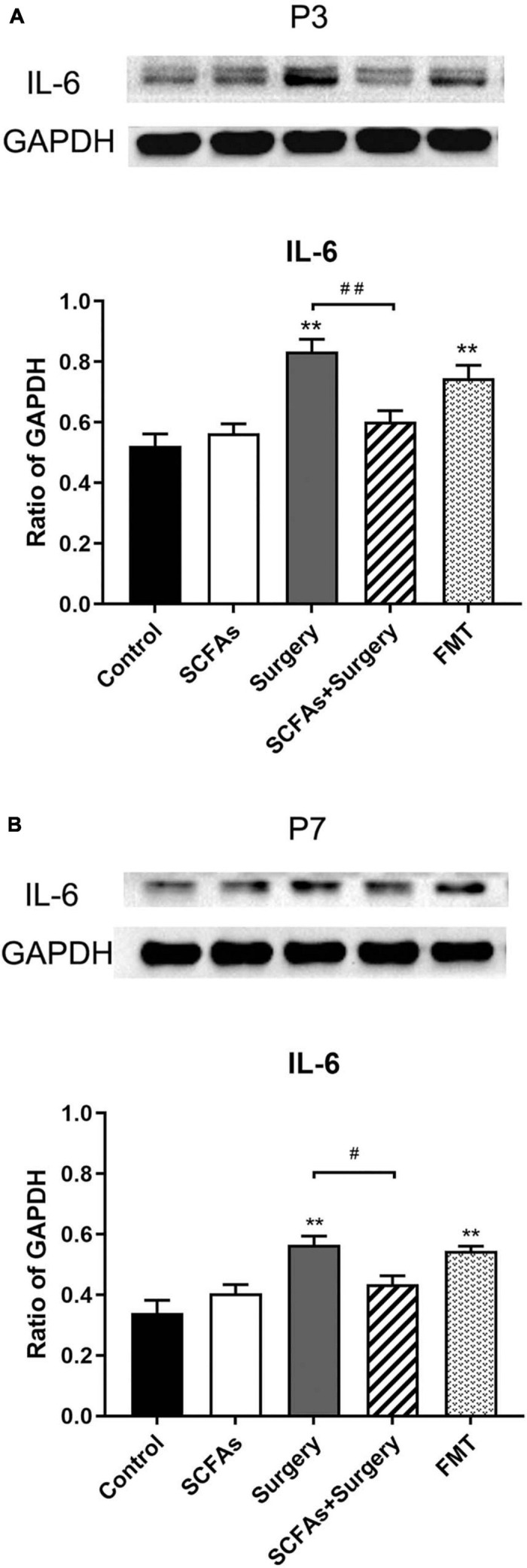
Surgical trauma and anesthesia and fecal microbiota transplantation significantly enhanced hippocampal IL-6 expression on postoperative or post-transplantation day 3 compared with the control group. Pretreatment with SCFAs partially reversed the higher expression of IL-6. The effects of SCFAs pretreatment for reversed IL-6 expression was maintained until day 7 compared with that of the surgery group. **(A)** The level of hippocampal IL-6 on postoperative or post-transplantation day 3. **(B)** The level of hippocampal IL-6 on postoperative or post-transplantation day 7. The results are represented as mean ± SEM. **p < 0.001 vs. the day-matched control group, ^#^*p* < 0.05 vs. the day-matched surgery group, ^##^*p* < 0.001 vs. the day-matched surgery group. P3 and P7: postoperative or post-transplantation days 3 and 7, respectively.

The protein levels of IL-1β and IL-6 in the hippocampus were significantly altered by surgery, exogenous SCFA administration, and days. There were no significant differences in protein levels between the control group and SCFA group. Compared with the controls, the levels of IL-1β and IL-6 were significantly increased on postoperative days 3 (*p* < 0.001, *p* < 0.001) and 7 (*p* = 0.001, *p* < 0.001) in the surgery group. Interestingly, compared with the control group, both IL-1β and IL-6 were significantly upregulated on post-transplantation days 3 and 7 in the FMT group (*p* = 0.001, *p* < 0.05, *p* < 0.001 and *p* < 0.001, respectively). Pretreatment with exogenous SCFAs partially decreased surgical trauma and anesthesia-induced upregulation of IL-1β and IL-6 in the hippocampus on postoperative day 3 (*p* < 0.05, *p* < 0.001) (Figures 4, 5).

### Surgical Trauma and Anesthesia Decreased the Production of SCFAs of Gut Microbes

**FIGURE 6:**
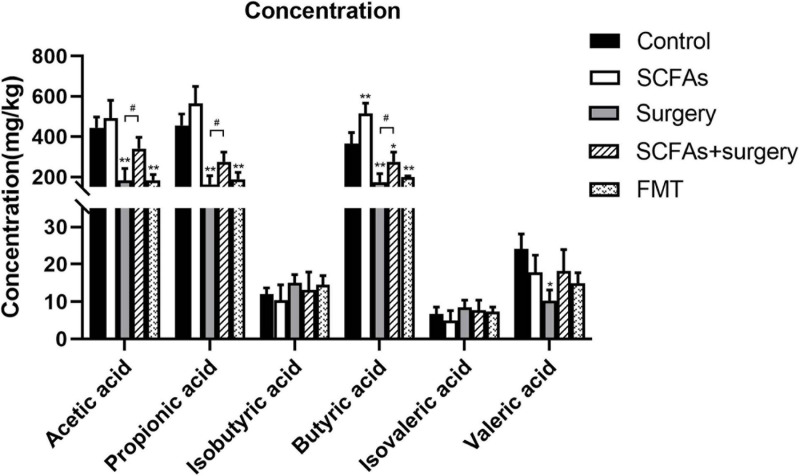
Surgical trauma and anesthesia and fecal microbiota transplantation from surgical mice significantly decreased the levels of straight-chain SCFAs on postoperative day 3 or post-transplantation day 3 compared with the controls. The concentration of straight-chain SCFAs in the surgery group showed significantly less than those in the SCFAs + surgery group. The results are represented as the mean ± SEM. *p < 0.05 vs. the control group, **p < 0.001 vs. the control group, ^#^*p* < 0.05 vs. the surgery group.

To investigate the effects of surgical trauma and anesthesia on the production of SCFAs of gut microbiota, the levels of SCFAs in feces were determined. The results of GC-MS revealed that surgical trauma, anesthesia, and fecal microbiota transplantation from surgical mice significantly decreased the levels of SCFAs, especially the straight-chain SCFAs, compared with the control group on postoperative day 3 (*p* < 0.001, *p* < 0.001). Notably, there was no significant difference in branched-chain SCFAs (BCFAs, e.g., isobutyric acid and isovaleric acid) (*p* > 0.05) (Figure 6).

### Surgical Trauma and Anesthesia Enhanced Microglial Activation, and Pretreatment With SCFAs Partially Reversed the Microglial Overactivation

**FIGURE 7:**
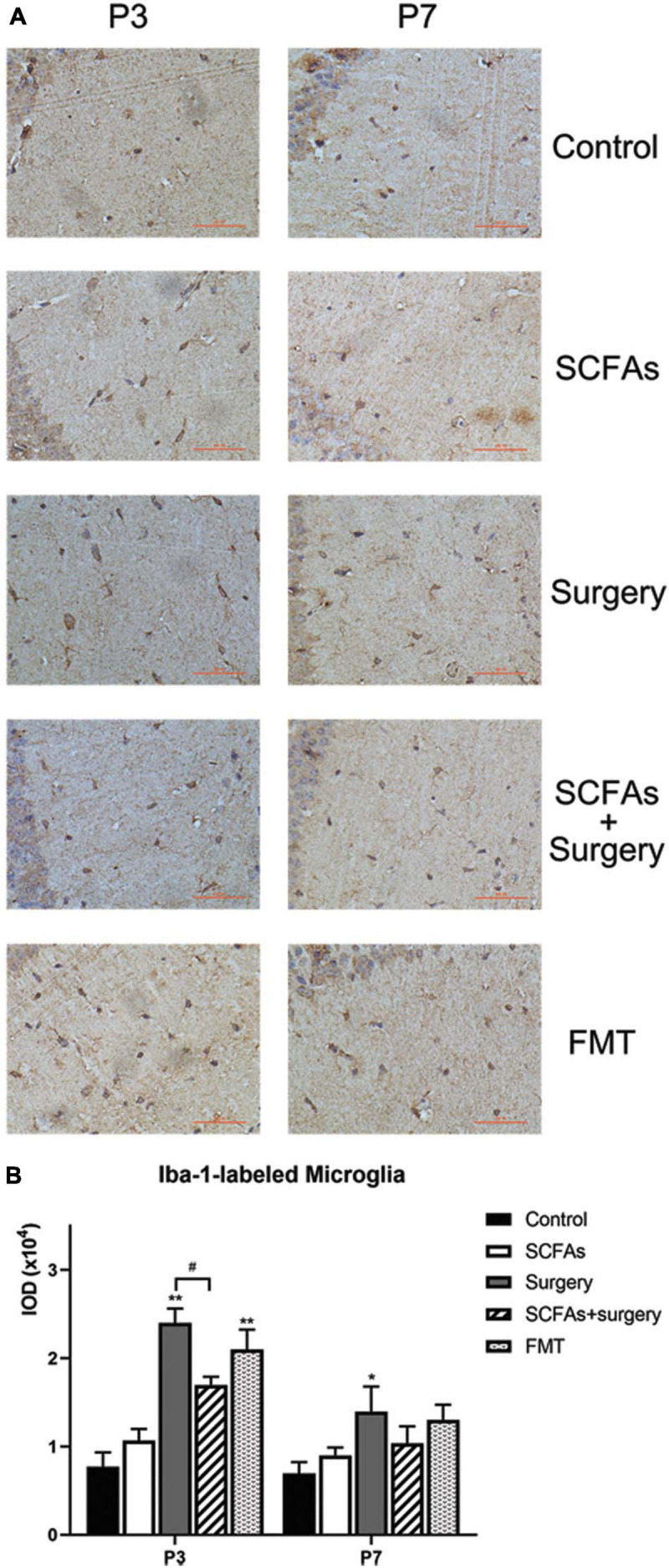
A significant difference for hippocampal Iba-1 levels was observed between control and surgical mice. Surgical trauma, anesthesia, and fecal microbiota transplantation enhanced Iba-1 expression and SCFA pretreatment partially reversed this change on postoperative day 3. **(A)** Representative images of Iba-1-labeled activated microglia in the hippocampus. **(B)** The IOD of Iba-1 in the hippocampus. The results are represented as mean ± SEM. *p < 0.05 vs. the day-matched control group, **p < 0.001 vs. the day-matched control group, ^#^*p* < 0.05 vs. the day-matched surgery group. P3 and P7: postoperative or post-transplantation days 3 and 7, respectively. Scale bars: 200 px.

A significant difference was observed for hippocampal Iba-1 levels by surgery, exogenous SCFA administration, and days (Figure 7A). Compared with the controls, administration with exogenous SCFAs alone failed to alter the levels of Iba-1 (*p* > 0.05). Surgical trauma and anesthesia significantly increased the levels of Iba-1 on postoperative day 3 (*p* < 0.001) and remained upregulated until postoperative day 7 (*p* < 0.001) compared with the control group. Fecal microbiota transplantation also significantly increased the levels of Iba-1 on post-transplantation day 3 (*p* < 0.05) and downregulated on post-transplantation day 7 (*p* > 0.05). Administration with exogenous SCFAs prior to surgery reduced the levels of Iba-1 in surgical mice on postoperative day 3 (*p* < 0.05) (Figure 7B).

### Surgical Trauma and Anesthesia Decreased the Levels of BDNF, and Administration of Exogenous SCFAs Prior to Surgery Enhanced BDNF Expression

**FIGURE 8:**
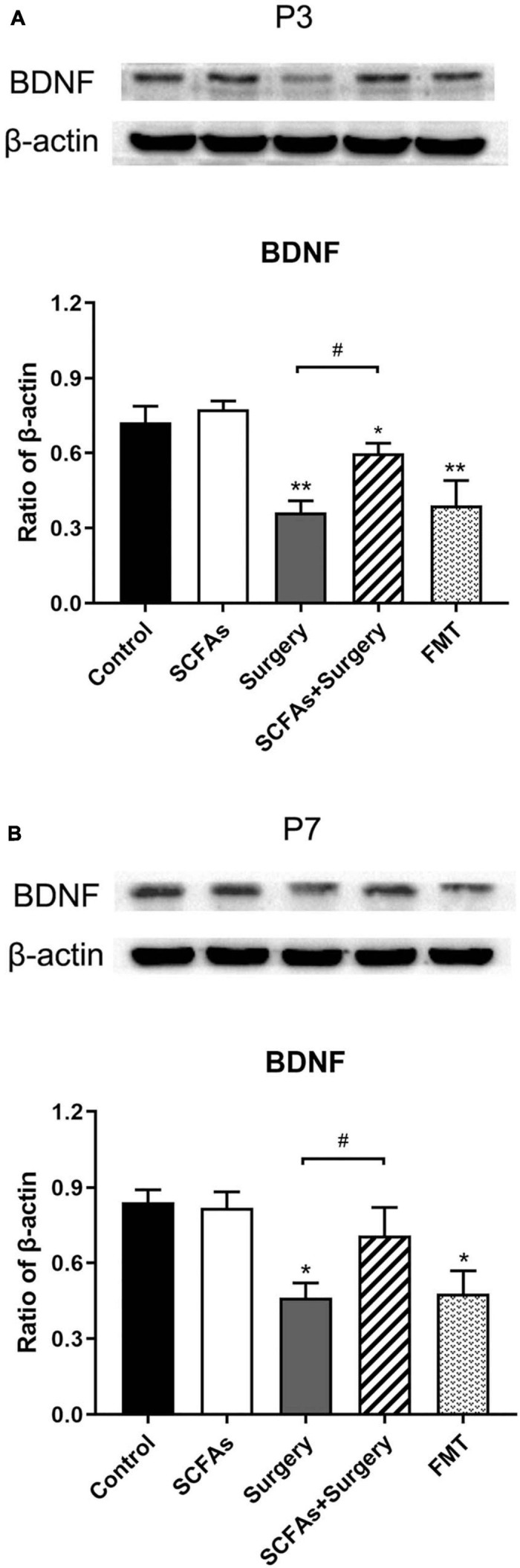
BDNF protein expression was significantly downregulated following surgery procedure and fecal microbiota transplantation compared with the nonsurgical control group on postoperative or post-transplantation day 3. Exogenous SCFA administration before surgery significantly upregulated the levels of BDNF compared with the surgery group on postoperative days 3 and 7. **(A)** The level of hippocampal BDNF on postoperative or post-transplantation day 3. **(B)** The level of hippocampal BDNF on postoperative or post-transplantation day 7. The results are represented as mean ± SEM. *p < 0.05 vs. the day-matched control group, **p < 0.001 vs. the day-matched control group, ^#^*p* < 0.05 vs. the day-matched surgery group. P3 and P7: postoperative or post-transplantation days 3 and 7, respectively.

Surgical trauma, anesthesia, and fecal microbiota transplantation from surgical mice significantly decreased the levels of BDNF compared with the controls on postoperative or post-transplantation days 3 (*p* < 0.001, *p* < 0.001) and 7 (*p* < 0.05, *p* < 0.05). Administration with exogenous SCFAs alone failed to upregulate the level of BDNF in normal mice (*p* > 0.05). However, exogenous SCFA treatment before surgery showed significant improvement over the surgery group on postoperative days 3 and 7 (*p* = 0.001, *p* < 0.05) (Figure 8).

## Discussion

This study demonstrates that surgical trauma exacerbates behavioral deficits and enhances neuroinflammatory responses in adult C57BL/6J mice. A surgery-induced decrease in SCFA expression coupled with exacerbated spatial learning and memory impairment reduced BDNF levels and amplified neuroinflammatory responses. Pretreatment with SCFAs attenuated these effects partially by reversing microglial overactivation, inhibiting neuroinflammatory responses, and enhancing BDNF expression. Therefore, SCFAs may be a potentially useful neuroprotective agent for preventing surgery-induced behavioral deficits.

The current study postulates that surgical trauma and anesthesia induced an exaggerated microglial activation and enhanced pro-inflammatory cytokine (IL-1β and IL-6) expression in the hippocampus of mice. The excessive inflammatory cytokine expression produces neurotoxicity responses: inhibition of remodeling of neural circuits and long-term potentiation (LTP) influence neuronal function either directly or through modulation of intraneuronal pathways (Li et al., 2017). SCFAs plays an important role in the modulation of microglial activation, and neuroinflammatory responses as well as surgical trauma and anesthesia induced behavioral deficits in mice (Li M. et al., 2018). Exogenous SCFA administration, at least partially, reversed surgical trauma and anesthesia induced spatial learning and memory impairment and inhibit neuroinflammatory responses in hippocampus. Cox et al. (2009) demonstrated that the increased production of SCFAs from dietary fiber supplementation or probiotics administration inhibited the production of proinflammatory mediators and recovered damaged colonic mucosa in colitic animals. Furthermore, fecal microbiota transplantation from surgical mice to normal pseudo germ-free mice resulted in spatial learning and memory impairments. These data indicate that impaired composition of gut microbiota is a contributor to the exaggerated microglial activation, neuroinflammatory responses, and behavioral deficits in response to surgery challenge.

Microbiota play a crucial role in regulating stress-related changes in brain function and behavior (Desbonnet et al., 2015; Burokas et al., 2017; Foster et al., 2017). Microbiota may help promote resilience against AD through multiple mechanisms, modulation of neuroinflammation, promotion of brain energy metabolism, and modulation of epigenetic mechanisms (Kelly et al., 2017). SCFAs may attenuate AD by interfering with the assembly of amyloid-β (Aβ) 1–40 and Aβ1–42 peptides into neurotoxic Aβ aggregates (Kelly et al., 2017). Yang et al. (2018) reported that PND could be alleviated using prebiotic galacto-oligosaccharide to target the gut–brain axis. Accumulating published evidence suggests that SCFAs can downregulate stress-signaling and hypothalamic–pituitary–adrenal (HPA) axis responsiveness, a crucial pathway in gut–brain–microbiota axis communication (Wiley et al., 2017). This study demonstrated that pretreatment with SCFAs partially reversed surgical trauma and anesthesia-induced behavioral deficits. Collectively, these findings suggest that exogenous supplementation with SCFAs may be beneficial for the prevention and treatment of PND.

Accumulating studies demonstrate that stress-related microbiota composition plays a causal role in behavioral changes (Maslowski et al., 2009; Mayer and Hsiao, 2017; Wiley et al., 2017; Misra and Mohanty, 2019; Zhan et al., 2019). Willing et al. (2011) demonstrated that fecal microbiota transplantation from stressor-exposed conventional mice to germ-free (GF) mice resulted in exaggerated inflammatory responses to Citrobacter rodentium infection. Consistent with the previous study, this experiment indicates that transplanting the microbiota from surgical mice to normal pseudo germ-free mice resulted in anxiety-related behavior and spatial learning and memory impairment, suggesting a direct role for microbiota composition in surgical trauma and anesthesia-induced behavioral deficits. These animal studies collectively highlight an association between altered gut microbiota and depressive-like behavior following surgery challenge.

Microglial dysfunction has been shown to be involved in the initiation or progression of multiple CNS diseases, including AD, depression, and autism spectrum disorder (Derecki et al., 2014; Shemer and Jung, 2015; Hong et al., 2016; Colonna and Butovsky, 2017; Voet et al., 2018; Giuliani et al., 2019). The gut microbiota, emerging as an important neuroimmunomodulator (Foster, 2016; Rea et al., 2016), is also involved in microglial maturation and activation (Cryan and Dinan, 2015). Recent studies provide important insights into the role of gut microbiota in microglial maturation, identity, and function, both in steady-state conditions and in diseases associated with elevated microglial activation (Cox et al., 2009; Cryan and Dinan, 2015). Limited microbiota complexity resulted in defective microglia. In contrast, recolonization with a complex microbiota partially restored microglial features. Recent evidence suggests that SCFAs modulate microglial maturation and function in the brain (Erny et al., 2015; Burokas et al., 2017), implicating the potential benefits of gut-derived SCFAs in modulating neuroinflammatory responses that play important roles across diverse neurodegenerative disorders, including AD. The decrease of SCFAs in surgical mice suggests that SCFAs may contribute to surgical trauma and anesthesia induced neuroinflammatory process. Oral administration of a mixture of three primary SCFAs (acetic acid, propionic acid, and butyric acid) for 4 weeks restored microglial overactivation and rescued surgical trauma and anesthesia induced behavioral deficits in this study. However, the exact SCFA signaling pathways that modulate microglial activation have yet to be fully elucidated.

BDNF is an important regulator of synaptic plasticity and LTP in the brain, which are involved in memory formation and maintenance. The gut microbiota plays a role in synapse maturation and synaptogenesis (Diaz Heijtz et al., 2011). BDNF gene expression is lower in the amygdala and cortex in male GF mice (Diaz Heijtz et al., 2011). Conversely, hippocampal BDNF levels are reduced in normal adult mice after they are exposed to an antibiotic regimen to deplete microbiota, which is reversed by a probiotic cocktail treatment (Möhle et al., 2016). Previous studies have demonstrated that severe stress suppresses BDNF signaling, impairs synaptic activity, and increases susceptibility to affective disorders. Furthermore, it has been suggested that exaggerated neuroinflammation may disrupt BDNF expression (Kernie et al., 2000). A study indicates that the levels of BDNF are also associated with SCFAs (Burokas et al., 2017). A growing body of evidence suggests that surgical trauma and anesthesia inhibit BDNF expression, resulting in cognitive impairment (Desbonnet et al., 2015). Consistent with the previous study, neuroinflammatory responses triggered by surgical trauma and anesthesia inhibited BDNF expression, which could be partially reversed by the administration of SCFAs.

Surgical trauma and anesthesia altered the composition of gut microbiota and induced behavioral deficits. Pretreatment with SCFAs to mice alleviates surgical trauma and anesthesia induced neuroinflammatory responses and spatial learning and memory impairment. Fecal microbiota transplantation from surgical mice aggravated performance on MWM and open-field tests in normal mice. These findings suggest that gut microbial-derived metabolite SCFAs are, at least partially, associated with behavior deficits in surgical mice. Taken together, these results present novel insights into the development of microbiota-targeted therapies for surgical trauma and anesthesia induced behavior deficits.

Some limitations of our study must be pointed out. Firstly, the alteration of the composition of bacteria in gut microbiota caused by surgery and anesthesia was not determined in the current study. We could not reveal which bacteria may be involved in surgery-induced behavior deficits. Secondly, postoperative behavior was evaluated only by the Morris water maze (MWM) and open-field tests. More tools are needed to determine behavioral performance in further study. Thirdly, the serum levels of SCFAs were not measured to reveal the difference of fecal and serum of SCFAs.

## Conclusion

Collectively, the current study provides evidence that gut microbiota are involved in spatial learning and memory impairment in mice caused by surgery and anesthesia. Its metabolites, SCFAs, partially reversed microglial overactivation, reduced the release of related inflammatory cytokine expression, and alleviated postoperative spatial learning and memory impairment.

## Data Availability Statement

The original contributions presented in the study are included in the article/supplementary material, further inquiries can be directed to the corresponding author/s.

## Ethics Statement

The animal study was reviewed and approved by the National Institutes of Health Guide for Care and Use of Laboratory Animals China Medical University Animal Care and Use Committee.

## Author Contributions

FL, XC, and XX conceived and designed the experiments. XX, KW, ZL, YZ, and JR performed the research and analyzed the data. XX wrote the manuscript. XC contributed to the manuscript revision. All the authors contributed to the article and approved the submitted version.

## Conflict of Interest

The authors declare that the research was conducted in the absence of any commercial or financial relationships that could be construed as a potential conflict of interest.

## References

[B1] Abdel-Haq R. Schlachetzki J. C. M. Glass C. K. Mazmanian S. K. (2019). Microbiome-microglia connections via the gut-brain axis. *J. Exp. Med.* 216 41–59. 10.1084/jem.20180794 30385457 PMC6314531

[B2] Block M. L. Zecca L. Hong J. S. (2007). Microglia-mediated neurotoxicity: uncovering the molecular mechanisms. *Nat. Rev. Neurosci.* 8 57–69. 10.1038/nrn2038 17180163

[B3] Bruce-Keller A. J. Salbaum J. M. Luo M. Blanchard E. III Taylor C. M. Welsh D. A. (2015). Obese-type gut microbiota induce neurobehavioral changes in the absence of obesity. *Biol. Psychiatry* 77 607–615. 10.1016/j.biopsych.2014.07.012 25173628 PMC4297748

[B4] Burokas A. Arboleya S. Moloney R. D. Peterson V. L. Murphy K. Clarke G. (2017). Targeting the microbiota-gut-brain axis: prebiotics have anxiolytic and antidepressant-like effects and reverse the impact of chronic stress in mice. *Biol. Psychiatry* 82 472–487. 10.1016/j.biopsych.2016.12.031 28242013

[B5] Cao X. Z. Ma H. Wang J. K. Liu F. Wu B. Y. Tian A. Y. (2010). Postoperative cognitive deficits and neuroinflammation in the hippocampus triggered by surgical trauma are exacerbated in aged rats. *Prog. Neuropsychopharmacol. Biol. Psychiatry* 34 1426–1432. 10.1016/j.pnpbp.2010.07.027 20691747

[B6] Colonna M. Butovsky O. (2017). Microglia function in the central nervous system during health and neurodegeneration. *Annu. Rev. Immunol.* 35 441–468. 10.1146/annurev-immunol-051116-052358 28226226 PMC8167938

[B7] Cox M. A. Jackson J. Stanton M. Rojas-Triana A. Bober L. Laverty M. (2009). Short-chain fatty acids act as antiinflammatory mediators by regulating prostaglandin E(2) and cytokines. *World J. Gastroenterol.* 15 5549–5557. 10.3748/wjg.15.5549 19938193 PMC2785057

[B8] Cryan J. F. Dinan T. G. (2015). Gut microbiota: microbiota and neuroimmune signalling-Metchnikoff to microglia. *Nat. Rev. Gastroenterol. Hepatol.* 12 494–496. 10.1038/nrgastro.2015.127 26215386

[B9] Derecki N. C. Katzmarski N. Kipnis J. Meyer-Luehmann M. (2014). Microglia as a critical player in both developmental and late-life CNS pathologies. *Acta Neuropathol.* 128 333–345. 10.1007/s00401-014-1321-z 25056803 PMC4131160

[B10] Desbonnet L. Clarke G. Traplin A. O’Sullivan O. Crispie F. Moloney R. D. (2015). Gut microbiota depletion from early adolescence in mice: implications for brain and behaviour. *Brain Behav. Immun.* 48 165–173. 10.1016/j.bbi.2015.04.004 25866195

[B11] Diaz Heijtz R. Wang S. Anuar F. Qian Y. Björkholm B. Samuelsson A. (2011). Normal gut microbiota modulates brain development and behavior. *Proc. Natl. Acad. Sci. U.S.A.* 108 3047–3052. 10.1073/pnas.1010529108 21282636 PMC3041077

[B12] Duca F. A. Sakar Y. Lepage P. Devime F. Langelier B. Doré J. (2014). Replication of obesity and associated signaling pathways through transfer of microbiota from obese-prone rats. *Diabetes* 63 1624–1636. 10.2337/db13-1526 24430437

[B13] Erny D. Hrabě de Angelis A. L. Jaitin D. Wieghofer P. Staszewski O. David E. (2015). Host microbiota constantly control maturation and function of microglia in the CNS. *Nat. Neurosci.* 18 965–977. 10.1038/nn.4030 26030851 PMC5528863

[B14] Foster J. A. (2016). Gut microbiome and behavior: focus on neuroimmune interactions. *Int. Rev. Neurobiol.* 131 49–65. 10.1016/bs.irn.2016.07.005 27793226

[B15] Foster J. A. Rinaman L. Cryan J. F. (2017). Stress & the gut-brain axis: regulation by the microbiome. *Neurobiol. Stress* 7 124–136. 10.1016/j.ynstr.2017.03.001 29276734 PMC5736941

[B16] Giuliani A. Sivilia S. Baldassarro V. A. Gusciglio M. Lorenzini L. Sannia M. (2019). Age-related changes of the neurovascular unit in the cerebral cortex of Alzheimer disease mouse models: a neuroanatomical and molecular study. *J. Neuropathol. Exp. Neurol.* 78 101–112. 10.1093/jnen/nly125 30629191

[B17] Hong S. Dissing-Olesen L. Stevens B. (2016). New insights on the role of microglia in synaptic pruning in health and disease. *Curr. Opin. Neurobiol.* 36 128–134. 10.1016/j.conb.2015.12.004 26745839 PMC5479435

[B18] Jiang Y. Li Z. Ma H. Cao X. Liu F. Tian A. (2018). Upregulation of TREM2 ameliorates neuroinflammatory responses and improves cognitive deficits triggered by surgical trauma in Appswe/PS1dE9 Mice. *Cell. Physiol. Biochem.* 46 1398–1411. 10.1159/000489155 29689568

[B19] Kelly J. R. Clarke G. Cryan J. F. Dinan T. G. (2016). Brain-gut-microbiota axis: challenges for translation in psychiatry. *Ann. Epidemiol.* 26 366–372. 10.1016/j.annepidem.2016.02.008 27005587

[B20] Kelly J. R. Kennedy P. J. Cryan J. F. Dinan T. G. Clarke G. Hyland N. P. (2015). Breaking down the barriers: the gut microbiome, intestinal permeability and stress-related psychiatric disorders. *Front. Cell. Neurosci.* 9:392. 10.3389/fncel.2015.00392 26528128 PMC4604320

[B21] Kelly J. R. Minuto C. Cryan J. F. Clarke G. Dinan T. G. (2017). Cross talk: the microbiota and neurodevelopmental disorders. *Front. Neurosci.* 11:490. 10.3389/fnins.2017.00490 28966571 PMC5605633

[B22] Kernie S. G. Liebl D. J. Parada L. F. (2000). BDNF regulates eating behavior and locomotor activity in mice. *EMBO J.* 19 1290–1300. 10.1093/emboj/19.6.1290 10716929 PMC305670

[B23] Li M. van Esch B. Wagenaar G. T. M. Garssen J. Folkerts G. Henricks P. A. J. (2018). Pro- and anti-inflammatory effects of short chain fatty acids on immune and endothelial cells. *Eur. J. Pharmacol.* 831 52–59. 10.1016/j.ejphar.2018.05.003 29750914

[B24] Li Z. Cao X. Ma H. Cui Y. Li X. Wang N. (2018). Surgical trauma exacerbates cognitive deficits and neuroinflammation in aged rats: the role of CX3CL1-CX3CR1 signaling. *J. Neuropathol. Exp. Neurol.* 77 736–746. 10.1093/jnen/nly051 29939299

[B25] Li Z. Liu F. Ma H. White P. F. Yumul R. Jiang Y. (2017). Age exacerbates surgery-induced cognitive impairment and neuroinflammation in Sprague-Dawley rats: the role of IL-4. *Brain Res.* 1665 65–73. 10.1016/j.brainres.2017.04.004 28414034

[B26] Lv F. Chen S. Wang L. Jiang R. Tian H. Li J. (2017). The role of microbiota in the pathogenesis of schizophrenia and major depressive disorder and the possibility of targeting microbiota as a treatment option. *Oncotarget* 8 100899–100907. 10.18632/oncotarget.21284 29246029 PMC5725071

[B27] Lyman M. Lloyd D. G. Ji X. Vizcaychipi M. P. Ma D. (2014). Neuroinflammation: the role and consequences. *Neurosci. Res.* 79 1–12. 10.1016/j.neures.2013.10.004 24144733

[B28] Maslowski K. M. Vieira A. T. Ng A. Kranich J. Sierro F. Yu D. (2009). Regulation of inflammatory responses by gut microbiota and chemoattractant receptor GPR43. *Nature* 461 1282–1286. 10.1038/nature08530 19865172 PMC3256734

[B29] Mayer E. A. Hsiao E. Y. (2017). The gut and its microbiome as related to central nervous system functioning and psychological well-being: introduction to the special issue of psychosomatic medicine. *Psychosom. Med.* 79 844–846. 10.1097/psy.0000000000000525 28976454 PMC5924442

[B30] Mazmanian S. K. Round J. L. Kasper D. L. (2008). A microbial symbiosis factor prevents intestinal inflammatory disease. *Nature* 453 620–625. 10.1038/nature07008 18509436

[B31] Misra S. Mohanty D. (2019). Psychobiotics: a new approach for treating mental illness? *Crit. Rev. Food Sci. Nutr.* 59 1230–1236. 10.1080/10408398.2017.1399860 29190117

[B32] Möhle L. Mattei D. Heimesaat M. M. Bereswill S. Fischer A. Alutis M. (2016). Ly6C(hi) monocytes provide a link between antibiotic-induced changes in gut microbiota and adult hippocampal neurogenesis. *Cell Rep.* 15 1945–1956. 10.1016/j.celrep.2016.04.074 27210745

[B33] Ni Y. F. Wang J. Yan X. L. Tian F. Zhao J. B. Wang Y. J. (2010). Histone deacetylase inhibitor, butyrate, attenuates lipopolysaccharide-induced acute lung injury in mice. *Respir. Res.* 11:33. 10.1186/1465-9921-11-33 20302656 PMC2848144

[B34] Rea K. Dinan T. G. Cryan J. F. (2016). The microbiome: a key regulator of stress and neuroinflammation. *Neurobiol. Stress* 4 23–33. 10.1016/j.ynstr.2016.03.001 27981187 PMC5146205

[B35] Rezaei Asl Z. Sepehri G. Salami M. (2019). Probiotic treatment improves the impaired spatial cognitive performance and restores synaptic plasticity in an animal model of Alzheimer’s disease. *Behav. Brain Res.* 376:112183. 10.1016/j.bbr.2019.112183 31472194

[B36] Shemer A. Jung S. (2015). Differential roles of resident microglia and infiltrating monocytes in murine CNS autoimmunity. *Semin. Immunopathol.* 37 613–623. 10.1007/s00281-015-0519-z 26240063

[B37] Smith P. M. Howitt M. R. Panikov N. Michaud M. Gallini C. A. Bohlooly Y. M. (2013). The microbial metabolites, short-chain fatty acids, regulate colonic Treg cell homeostasis. *Science* 341 569–573. 10.1126/science.1241165 23828891 PMC3807819

[B38] Steinmetz J. Christensen K. B. Lund T. Lohse N. Rasmussen L. S. (2009). Long-term consequences of postoperative cognitive dysfunction. *Anesthesiology* 110 548–555. 10.1097/ALN.0b013e318195b569 19225398

[B39] van de Wouw M. Boehme M. Lyte J. M. Wiley N. Strain C. O’Sullivan O. (2018). Short-chain fatty acids: microbial metabolites that alleviate stress-induced brain-gut axis alterations. *J. Physiol.* 596 4923–4944. 10.1113/jp276431 30066368 PMC6187046

[B40] Vinolo M. A. Rodrigues H. G. Hatanaka E. Sato F. T. Sampaio S. C. Curi R. (2011). Suppressive effect of short-chain fatty acids on production of proinflammatory mediators by neutrophils. *J. Nutr. Biochem.* 22 849–855. 10.1016/j.jnutbio.2010.07.009 21167700

[B41] Voet S. Mc Guire C. Hagemeyer N. Martens A. Schroeder A. Wieghofer P. (2018). A20 critically controls microglia activation and inhibits inflammasome-dependent neuroinflammation. *Nat. Commun.* 9:2036. 10.1038/s41467-018-04376-5 29789522 PMC5964249

[B42] Wang N. Ma H. Li Z. Gao Y. Cao X. Jiang Y. (2017). Chronic unpredictable stress exacerbates surgery-induced sickness behavior and neuroinflammatory responses via glucocorticoids secretion in adult rats. *PLoS One* 12:e0183077. 10.1371/journal.pone.0183077 28806788 PMC5555668

[B43] Wang Y. Cao X. Ma H. Tan W. Zhang L. Li Z. (2016). Prior stressor exposure delays the recovery of surgery-induced cognitive impairment and prolongs neuroinflammation in aged rats. *Brain Res.* 1648(Pt. A) 380–386. 10.1016/j.brainres.2016.07.045 27487302

[B44] Wen L. Ley R. E. Volchkov P. Y. Stranges P. B. Avanesyan L. Stonebraker A. C. (2008). Innate immunity and intestinal microbiota in the development of Type 1 diabetes. *Nature* 455 1109–1113. 10.1038/nature07336 18806780 PMC2574766

[B45] Wiley N. C. Dinan T. G. Ross R. P. Stanton C. Clarke G. Cryan J. F. (2017). The microbiota-gut-brain axis as a key regulator of neural function and the stress response: implications for human and animal health. *J. Anim. Sci.* 95 3225–3246. 10.2527/jas.2016.1256 28727115

[B46] Willing B. P. Vacharaksa A. Croxen M. Thanachayanont T. Finlay B. B. (2011). Altering host resistance to infections through microbial transplantation. *PLoS One* 6:e26988. 10.1371/journal.pone.0026988 22046427 PMC3203939

[B47] Yang C. Fujita Y. Ren Q. Ma M. Dong C. Hashimoto K. (2017a). Bifidobacterium in the gut microbiota confer resilience to chronic social defeat stress in mice. *Sci. Rep.* 7:45942. 10.1038/srep45942 28368029 PMC5377462

[B48] Yang C. Qu Y. Fujita Y. Ren Q. Ma M. Dong C. (2017b). Possible role of the gut microbiota-brain axis in the antidepressant effects of (R)-ketamine in a social defeat stress model. *Transl. Psychiatry* 7:1294. 10.1038/s41398-017-0031-4 29249803 PMC5802627

[B49] Yang X. D. Wang L. K. Wu H. Y. Jiao L. (2018). Effects of prebiotic galacto-oligosaccharide on postoperative cognitive dysfunction and neuroinflammation through targeting of the gut-brain axis. *BMC Anesthesiol.* 18:177. 10.1186/s12871-018-0642-1 30497394 PMC6267821

[B50] Yu F. Han W. Zhan G. Li S. Xiang S. Zhu B. (2019). Abnormal gut microbiota composition contributes to cognitive dysfunction in streptozotocin-induced diabetic mice. *Aging (Albany N. Y.)* 11 3262–3279. 10.18632/aging.101978 31123221 PMC6555457

[B51] Zhai S. Zhu L. Qin S. Li L. (2018). Effect of lactulose intervention on gut microbiota and short chain fatty acid composition of C57BL/6J mice. *Microbiologyopen* 7:e00612. 10.1002/mbo3.612 29575825 PMC6291785

[B52] Zhan G. Hua D. Huang N. Wang Y. Li S. Zhou Z. (2019). Anesthesia and surgery induce cognitive dysfunction in elderly male mice: the role of gut microbiota. *Aging (Albany N. Y.)* 11 1778–1790. 10.18632/aging.101871 30904902 PMC6461176

[B53] Zhan G. Yang N. Li S. Huang N. Fang X. Zhang J. (2018). Abnormal gut microbiota composition contributes to cognitive dysfunction in SAMP8 mice. *Aging (Albany N. Y.)* 10 1257–1267. 10.18632/aging.101464 29886457 PMC6046237

[B54] Zheng P. Zeng B. Zhou C. Liu M. Fang Z. Xu X. (2016). Gut microbiome remodeling induces depressive-like behaviors through a pathway mediated by the host’s metabolism. *Mol. Psychiatry* 21 786–796. 10.1038/mp.2016.44 27067014

[B55] Zhou Y. Li Z. Cao X. Ma H. White P. F. Xu X. (2019). Exendin-4 improves behaviorial deficits via GLP-1/GLP-1R signaling following partial hepatectomy. *Brain Res.* 1706 116–124. 10.1016/j.brainres.2018.11.007 30408479

